# Supplementary material for: Reconfigurable hyperbolic polaritonics with correlated oxide metasurfaces
Source: Nat Commun. 2022 Aug 3;13:4511. doi: 10.1038/s41467-022-32287-z (PMC9349304; doi:10.1038/s41467-022-32287-z)
Supplement: Supplementary file 1 — Supplementary Information [file 41467_2022_32287_MOESM1_ESM.pdf]

Supplementary information

Reconfigurable Hyperbolic Polaritonics with Correlated Oxide Metasurfaces

Neda Alsadat Aghamiri[1], Guangwei Hu[2,3], Alireza Fali[1], Zhen Zhang[4], Jiahan Li[5], Sivacarendran Balendhran[6], Sumeet Walia[7,8], Sharath Sriram[8,9], James H. Edgar[5], Shriram Ramanathan[4], Andrea Alù[2,10], Yohannes Abate[1*]

1 Department of Physics and Astronomy, University of Georgia, Athens, Georgia 30602, USA

2 Photonics Initiative, Advanced Science Research Center, City University of New York, New York, NY 10031, USA

3 Department of Electrical and Computer Engineering, National University of Singapore, Kent Ridge, Singapore 117583, Singapore

4 School of Materials Engineering, Purdue University, West Lafayette, IN 47907, USA

5 Tim Taylor Department of Chemical Engineering, Kansas State University, Manhattan, KN 66506, USA

6 School of Physics, University of Melbourne, Parkville, Victoria 3010, Australia

7 School of Engineering RMIT University Melbourne, Victoria, Australia

8 Functional Materials and Microsystems Research Group and the Micro Nano Research Facility RMIT University Melbourne, Victoria, Australia

9 ARC Centre of Excellence for Transformative Meta-Optical Systems, RMIT University, Melbourne, Victoria, Australia

10 Physics Program, Graduate Center, City University of New York, New York, NY 10016, USA

e-mail: yohannes.abate@uga.edu

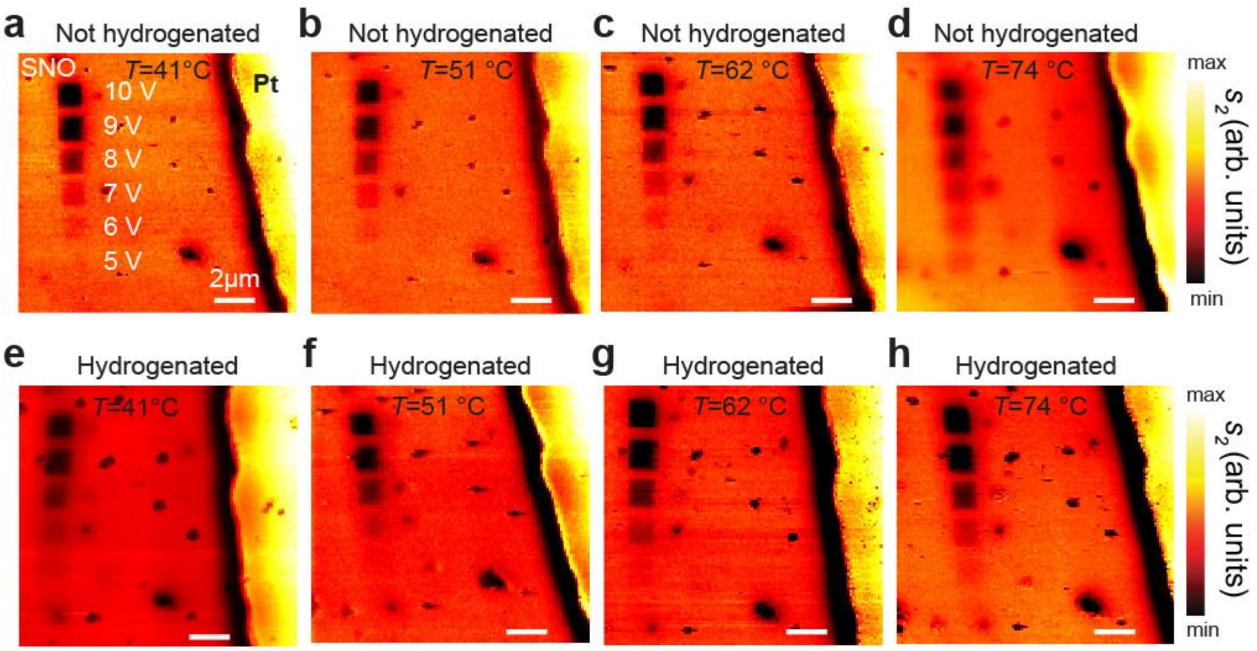

Supplementary Figure 1 a-d Second harmonic near field amplitude s_2 images at different temperatures (no H_2 exposure). **e-h** Second harmonic near field amplitude s_2 images at different temperatures after exposing to 5% H_2 gas while heating at T=100 °C all imaged at λ= 10.5 µm.

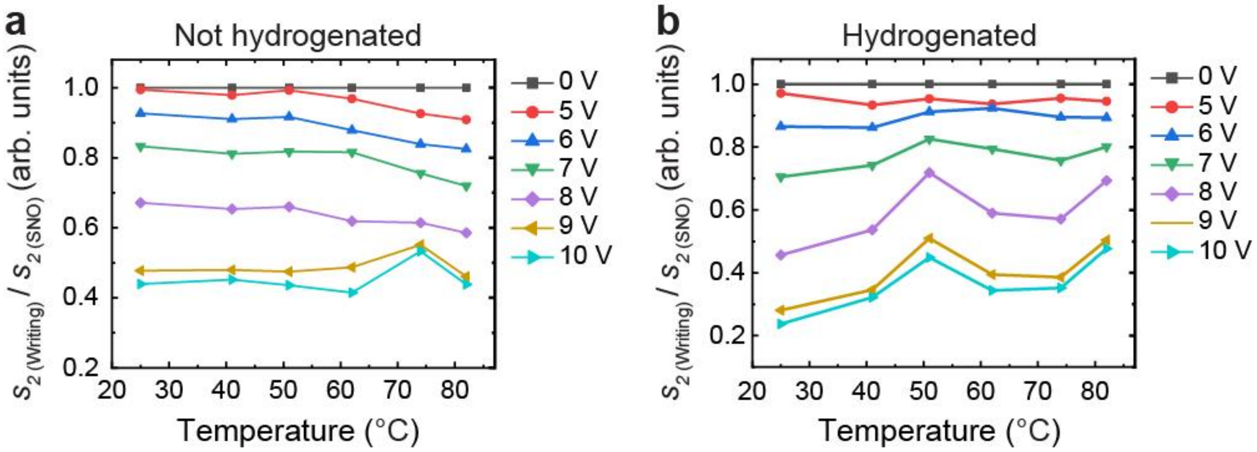

Supplementary Figure 2 a Normalized amplitude $s_{2\ (\mathrm{writing})}$ /$s_{2\ (\mathrm{SNO})}$ without exposing to H_2 for different temperatures. **b** Normalized amplitude $s_{2\ (\mathrm{writing})}$ / $s_{2\ (\mathrm{SNO})}$ after being exposed to 5% H_2 gas while heating at 100 °C for different temperatures. Each normalized amplitude points on these plots were extracted by averaging 3 × 3 pixels of the s-SNOM signal at the middle of each square pattern followed by normalization with signal on Pt electrode. Each normalized amplitude points on these plots were extracted by averaging 3 × 3 pixels of the s-

SNOM signal at the middle of each square pattern followed by normalization with signal on Pt electrode. The bumps shown in panel b at 50 °C could be caused due to the written squares not being uniformly insulating affecting the temperature dependent s-SNOM signal.

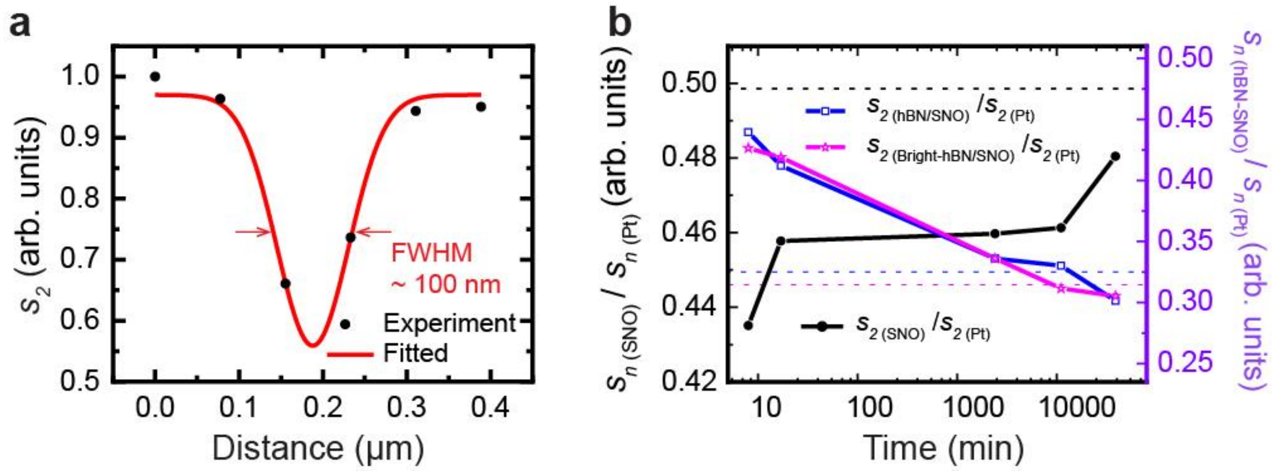

Supplementary Figure 3 a Near-field amplitude s_2 line profile extended along the black dashed line in Fig. 2f (black dots) and the fitted plot (solid red) showing FWHM~ 100 nm for a typical square. **b** Normalized amplitude plots of s_n (SNO) /s_n (Pt) as a function of time after hydrogen doping for SNO surface with no hBN (left *y*-axis), the right *y*-axis shows normalized amplitude plot taken on the surface of heterostructure hBN/SNO (blue points) and normalized amplitude plot taken on the nano-resonator (Bright-hBN/SNO) (pink points) as a function of time after hydrogen doping.

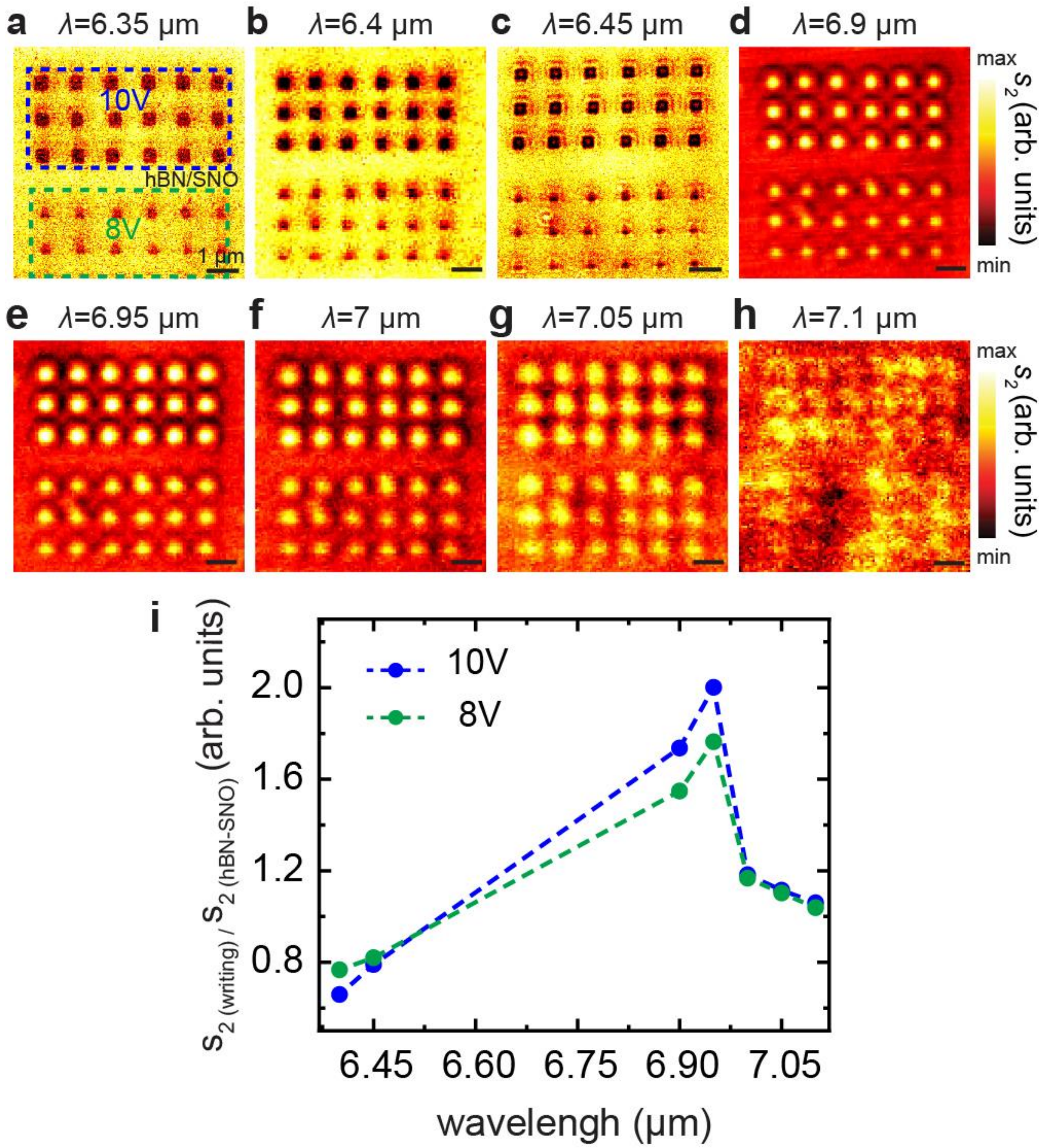

Supplementary Figure 4 S-SNOM second harmonic near-field amplitude, s_2, images of pristine SNO with conductive writing patterns made by applying potential at the c-AFM tip, 10 V (three top rows) and 8 V (three bottom rows in a-h) with a hBN flake transferred on top of the pattern taken at laser excitation wavelength, **a** λ=6.35 μm**,** **b** λ =6.4 μm, **c** λ =6.45 μm , **d** λ =6.9 μm, **e** λ =6.95 μm, **f** λ =7 μm , **g** λ =7.05 μm, **h** λ =7.1 μm . **i** Normalized amplitude intensity plots of nano-confined hot-spots $s_{2\,(\mathrm{writing})}$ /$s_{2\,(\mathrm{hBN/SNO})}$ as a function of wavelength. All scale bars indicate 1 μm.

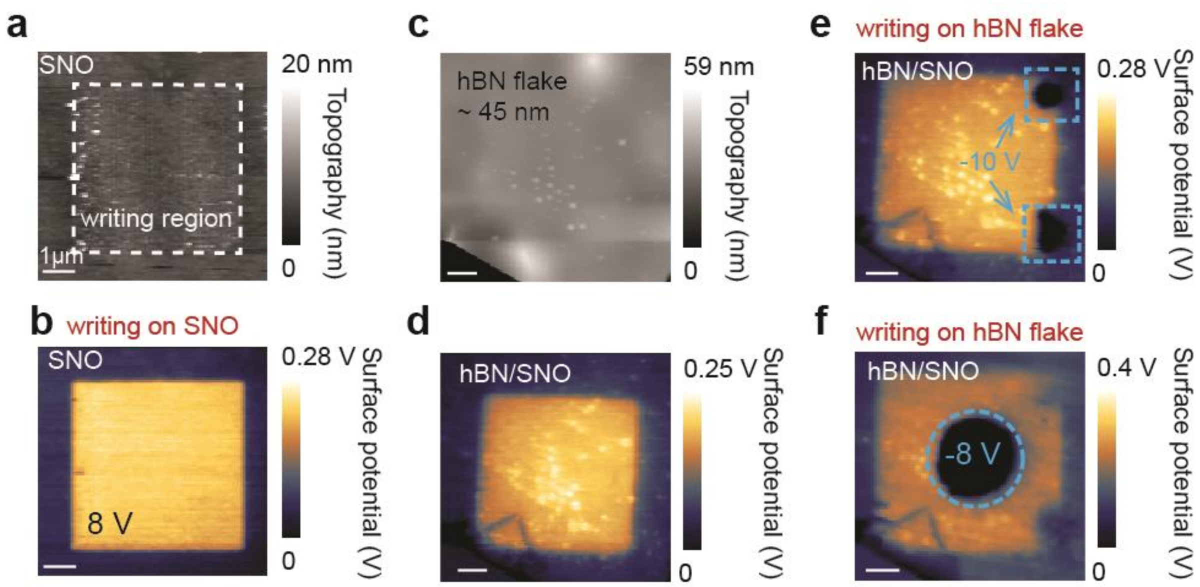

Supplementary Figure 5 a Topography of SNO surface showing the writing region. **b** SKPM surface potential image of conductive writing patterns on pristine SNO (shown in a) made by applying 8 V potential at the c-AFM tip. **c** Topography of hBN transferred on SNO with a writing (shown in Supplementary Figure 5a and b), **d** SKPM image of the hBN/SNO surface shown in c revealing the conductive writing pattern below hBN. **e** Conductive writing patterns on top of hBN/SNO made by applying -10V potential to the c-AFM tip (cyan dashed boxes). **f** Conductive writing patterns on hBN/SNO made by applying -8V potential at the c-AFM tip (cyan dashed circle).

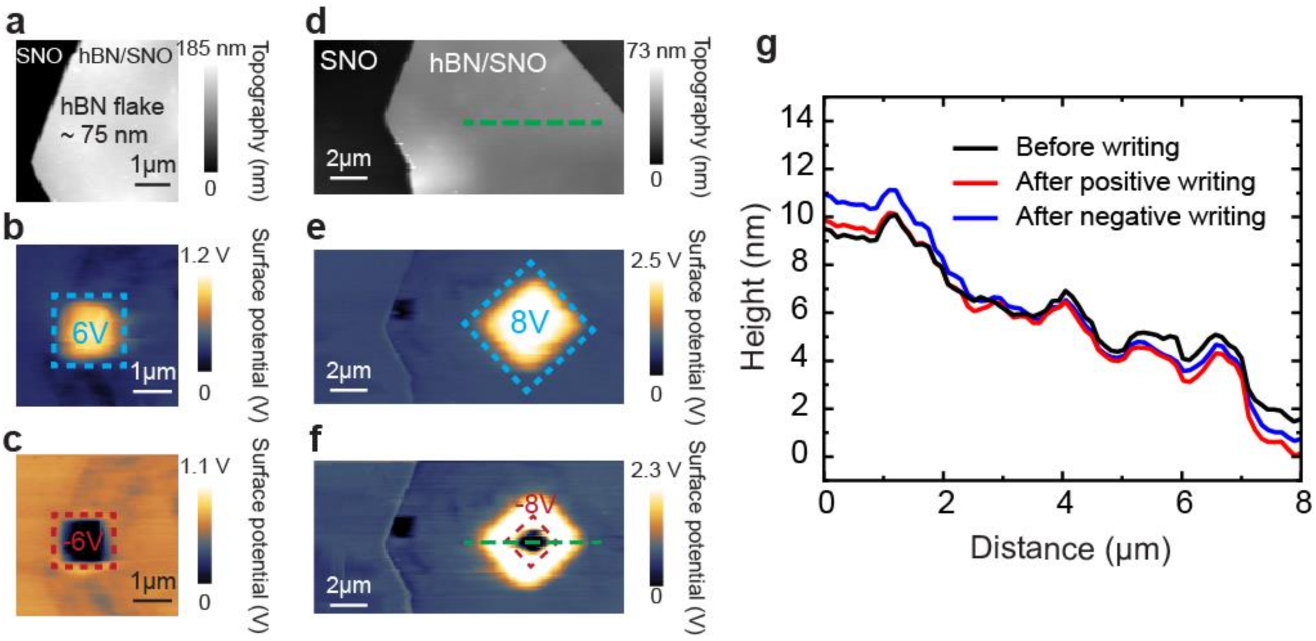

Supplementary Figure 6 a Topography of hBN on pristine SNO. **b** SKPM surface potential image of conductive patterns written on top of hBN/SNO made by applying 6V potential to the c-AFM tip (cyan dashed box). **c** SKPM surface potential image made by applying -6V potential (red dashed box) on the same region as b (effectively erasing of potential shown in b). **d** Larger topography image of the same flake shown in a. **e** SKPM surface potential image of a conductive larger square patterns written on top of hBN/SNO made by applying 8 V potential at the c-AFM tip (cyan dashed box), **f** SKPM image of the same region as e but a concentric erasing shown in the middle made by applying -8V potential at the c-AFM tip (red dashed box). **g** Topography line profile taken on d (shown by the green broken line) also shown in f. No meaningful topographic change (within the *z*-axis resolution of our AFM) has been observed on hBN surface.

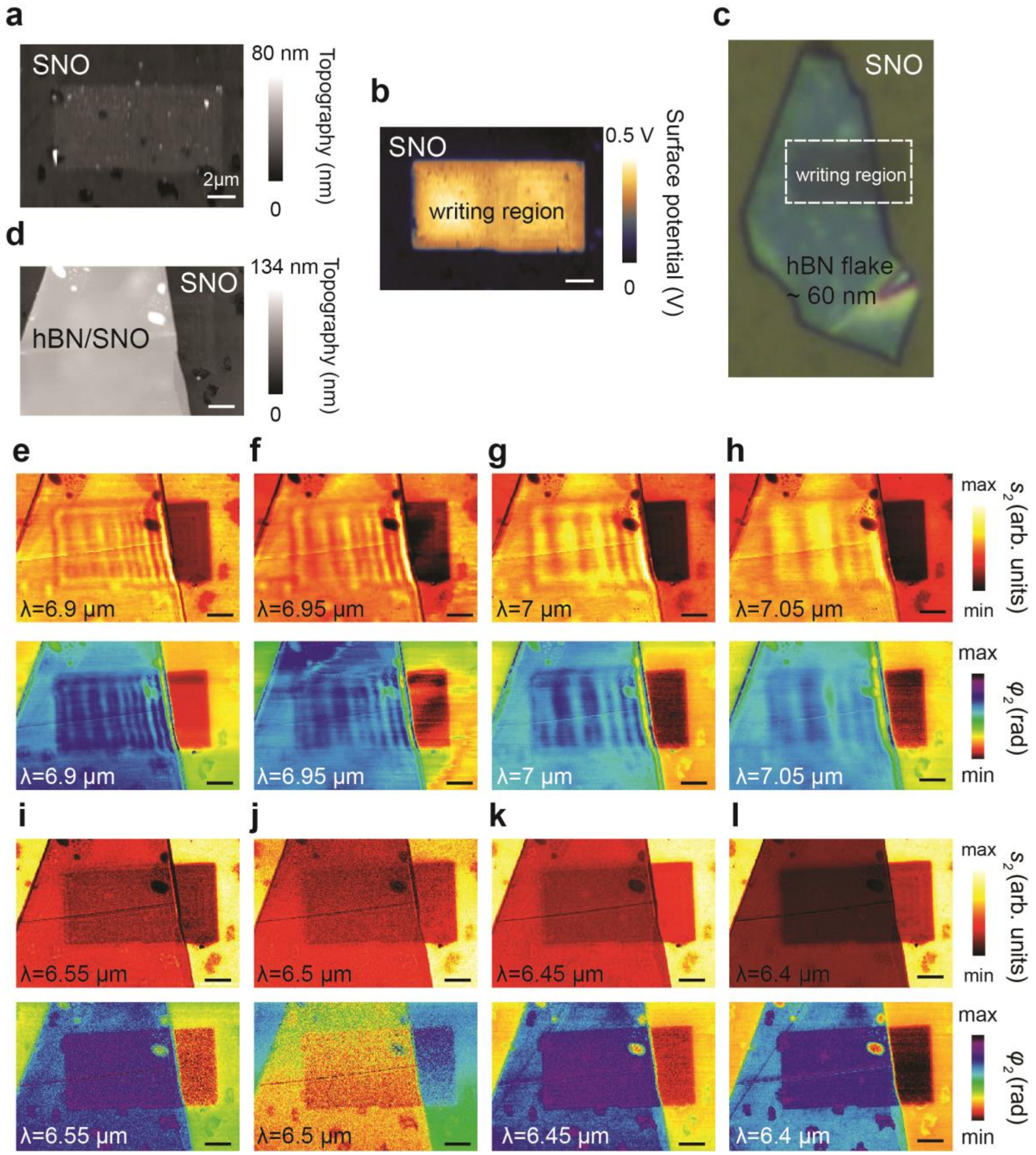

Supplementary Figure 7 **a** Topography, **b** Surface potential showing the lithography pattern made with SPM using 10 V. **c** Picture of the flake transferred on the top of SNO including writing pattern of part b. **d** Topography image of the pristine SNO with transferred hBN flake on the top. Corresponding second-harmonic near field amplitude s_2 and phase φ_2 images of part d at **e** 6.9 µm, **f** 6.95 µm, **g** 7 µm, **h** 7.05 µm, **i** 6.55 µm, **j** 6.5 µm, **k** 6.45 µm and **l** 6.9 µm. All scale bars indicate 2 µm.

Supplementary Note 1

In-plane anisotropic propagation of polaritons can be realized by either exploiting natural in-plane anisotropic crystals [1,2] or structuring in-plane isotropic crystals to metasurfaces [3,4]. The later method however requires demanding fabrication techniques and suffers from nonlocality [5]. In Supplementary Figure 8 we counterintuitively propose and demonstrate anisotropic propagation of polaritons in pristine in-plane isotropic crystal, via changing part of insulator (positive written area) SNO substrate to pristine phase, forming the SNO grating (SNO-G). As shown in Supplementary Figure 8a, we fabricate a hBN/SNO-G heterostructure, where the width of SNO in insulator (positive written area) and pristine phase are (W_1=) 132 and (W_2=) 160 nm. Supplementary Figures 8b-c show s-SNOM second harmonic amplitude images of propagating polaritons taken at 6.9 µm and 6.95 µm laser wavelengths. The propagation of polaritons perpendicular to the grating is easily visible while the polariton propagation along the SNO ribbon is less obvious, as shown in Supplementary Figure 8f. This provides a clear signature of anisotropic propagation of polaritons which resulted due to the anisotropic patterns in SNO.

To model our system, we could homogenize deep-wavelength grating beneath with average medium theory, which is written as $\varepsilon_{\parallel} = \rho\varepsilon_1 + (1-\rho)\varepsilon_2$; $\varepsilon_{\perp} = \left(\frac{\rho}{\varepsilon_1} + \frac{1-\rho}{\varepsilon_2}\right)^{-1}$. Here, $\rho = \frac{W_1}{W_1+W_2}$, ε_1 and ε_2 are the permittivity of SNO in insulator (h-doped or positive written area) and pristine phase[6]; $\varepsilon_{\parallel}$ and $\varepsilon_{\perp}$ are the effective permittivity of substrate parallel and perpendicular to the SNO-G, which are plot in Supplementary Figure 8d. In that sense, we construct an anisotropic-isotropic interface that could tune the polaritonic response in hBN. The dispersion of hBN polaritons along two principal directions (∥ and ⊥) is calculate by

$$k_j = -\frac{\Phi}{d}\left[\operatorname{atan}\left(\frac{\varepsilon_0}{\varepsilon_{hBN,\perp}}\Phi\right) + \operatorname{atan}\left(\frac{\varepsilon_j}{\varepsilon_{hBN,\perp}}\Phi\right) + l\pi\right] \quad (1)$$

where $\Phi = \sqrt{-\frac{\varepsilon_{hBN,\parallel}}{\varepsilon_{hBN,\perp}}}$, ε_j is $\varepsilon_{\parallel}$ for momentum along SNO-G direction and is $\varepsilon_{\perp}$ perpendicular the SNO-G direction; $\varepsilon_{hBN,\perp}$ and $\varepsilon_{hBN,\parallel}$ are permittivity of hBN perpendicular and parallel to OA; and l is the mode index. The calculated dispersion is provided in Supplementary Figure 8e, which shows strong difference of momentum along and perpendicular to the SNO-G, justifying the anisotropic propagation

of hBN. This result provides an alternative avenue towards anisotropic propagation of polaritons, totally different from all previous methods, as it tailors the substrate's anisotropy. Nevertheless, since the spatial resolution of tunable substrate can be further enhanced by our PCM techniques and other pattern can be directly applied, we expect more degree of freedoms to control the polaritons and more reconfigurable functional metamaterials devices can be developed from this technique [7].

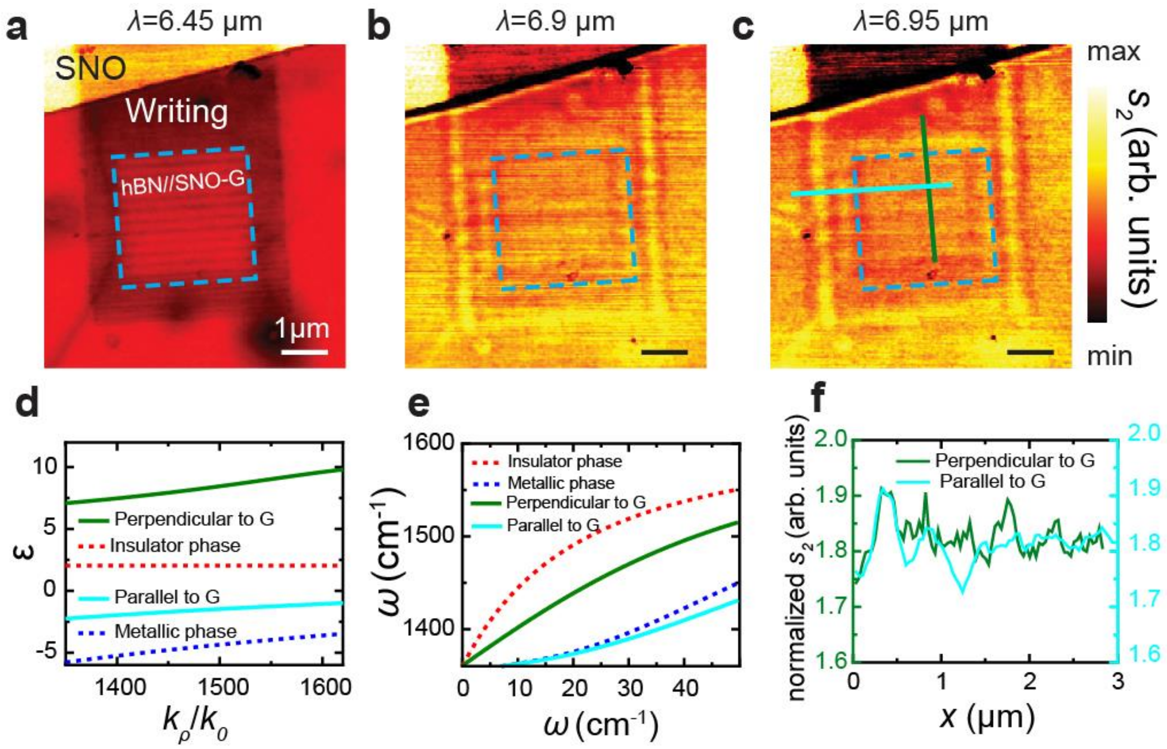

Supplementary Figure 8 Anisotropic propagation of polaritons in hBN. IR s-SNOM second harmonic near field amplitude s_2 images of hBN/SNO-G (Grating) at **a** 6.45 μm, **b** 6.9 μm and **c** 6.95 μm, scale bar is 2 μm. **d** Amplitude s_2 line profile as indicated in panel c with green and blue lines showing polariton propagating parallel and perpendicular to the grating, the red dashed line shows where grating starts. **e** The effective permittivity of substrate, the red, blue, cyan and green solid lines represent the permittivity of SNO in insulator phase (positive written area), SNO in pristine phase, SNO-G parallel to ribbon and SNO-G perpendicular to ribbon. **f** The calculated polariton momentum with the substrate of insulator SNO (red dashed line), pristine SNO (blue dashed line), along SNO-G (cyan solid line) and perpendicular to SNO-G directions.

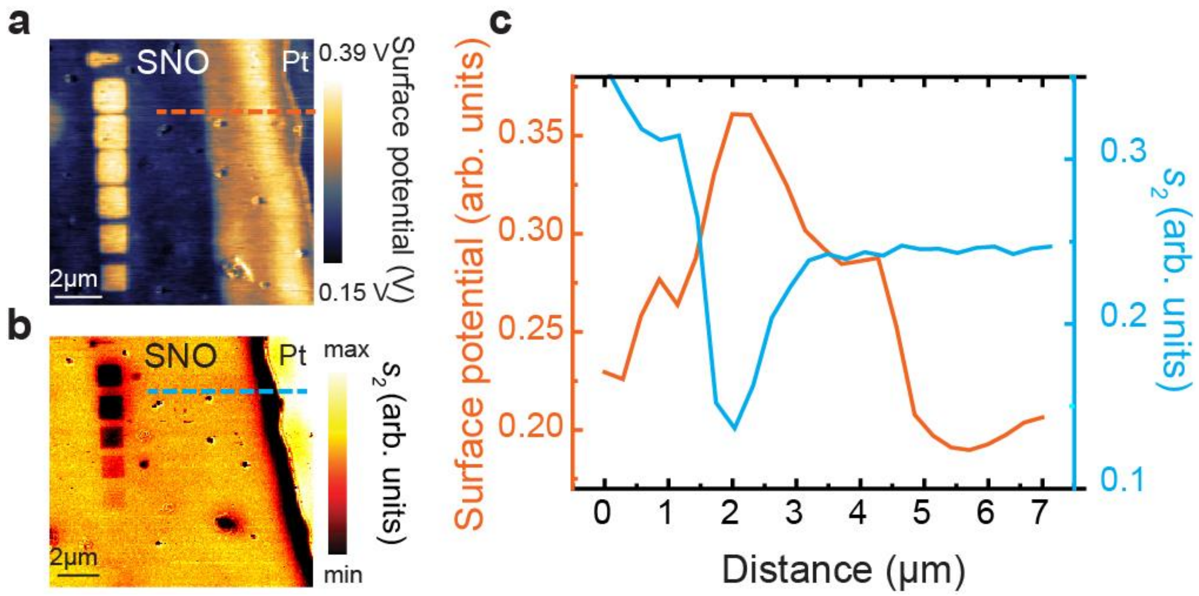

Supplementary Figure 9 a SKPM surface potential image of conductive writing patterns on pristine SNO made by applying V=5 V-10 V potential at the c-AFM tip. **b** Corresponding infrared s-SNOM second harmonic near-field amplitude s_2 image at T=23 °C (no H_2 exposure), imaged at λ=10.5 μm. **c** plots showing the surface potential line profile extended along the orange dashed line in panel a (left axis) and second harmonic near-field amplitude s_2 line profile extended along the blue dashed line in panel b (right axis).

Supplementary Note 2

The bright region in the SKPM image in Supplementary Figure 9a at the right of the sample indicates an insulating region near the edge of the Pt is. While the s-SNOM image (shown in dark contrast near the Pt edge in Supplementary Figure 9b) shows parts of the extended insulating region. Supplementary Figure 9c Show the line profiles taken at the SKPM image (orange line) and the near-field image (blue line). Not all of the extended insulating region shown in the SKPM is picked up by the s-SNOM due to difference in sensitivity.

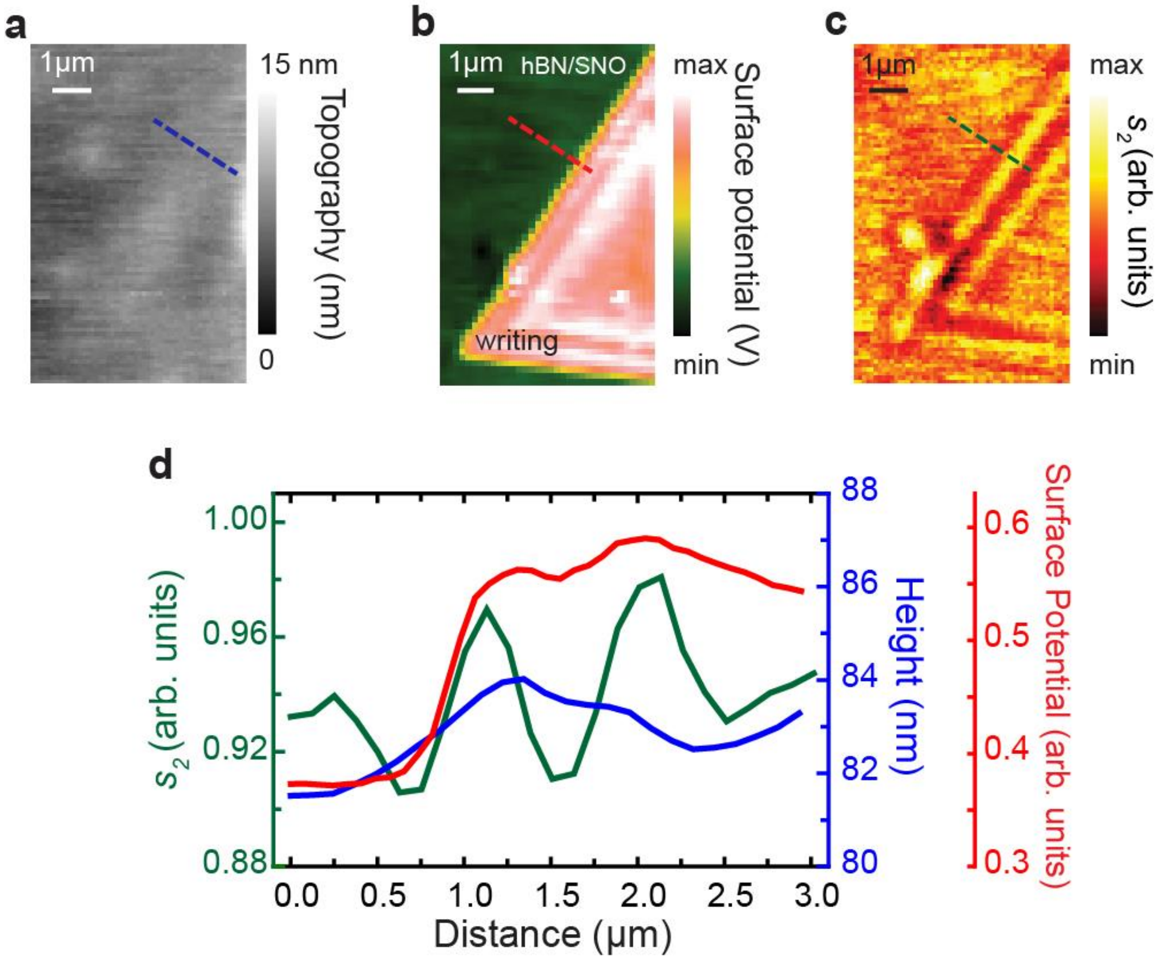

Supplementary Figure 10 Zoomed-in image of Fig. 4d and 4e of the main manuscript to illustrate origin of polariton fringes. a AFM topography and **b** SKPM surface potential image of conductive writing patterns on pristine SNO made by applying 10V potential at the c-AFM tip. **c** Corresponding IR s-SNOM second harmonic near-field amplitude s_2 images at T=23 °C (no H_2 exposure). **d** Line profile plot of the topography (blue line) extended along the blue dashed line in panel a, SKPM line profile (red line) extended along the

red dashed line in panel b and second harmonic near-field amplitude s_2 line profile (green line) extended along the green dashed line in panel a.

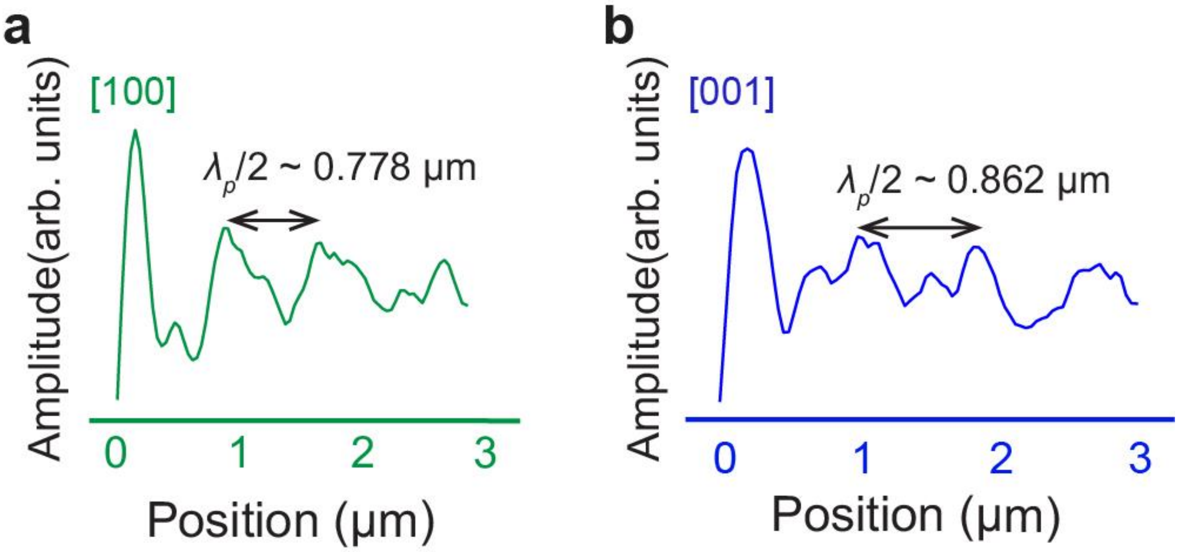

Supplementary Figure 11 Enlarged image of the inset in Fig. 5a of the main manuscript. a Line profile along the [100] in Fig. 5a showing the polariton propagation perpendicular to the edge of α-MoO_3 flake. **b** Line profile along the [100] in Fig. 5a showing the polariton propagation parallel to the edge of α-MoO_3 flake.

Supplementary Note 3

Supplementary Figure 12 a-c show the same near-field images as Fig. 5a-c but with broken lines added along the [100] and [001] axis. Like in Supplementary Figure 11 the line profile along the [100] shows a shorter wavelength of the polariton compared with the one along the [100] (polariton propagation parallel to the edge of α-MoO_3 flake). A similar, although weak, axis dependence of the polariton wavelength is also plotted at 10.76 μm (orange line in d and light blue line in e. The line profiles in either axis in Fig 4b (Supplementary Figure 12 b) are flat due to the absence of polariton at 10.25 μm (green lines in d and e).

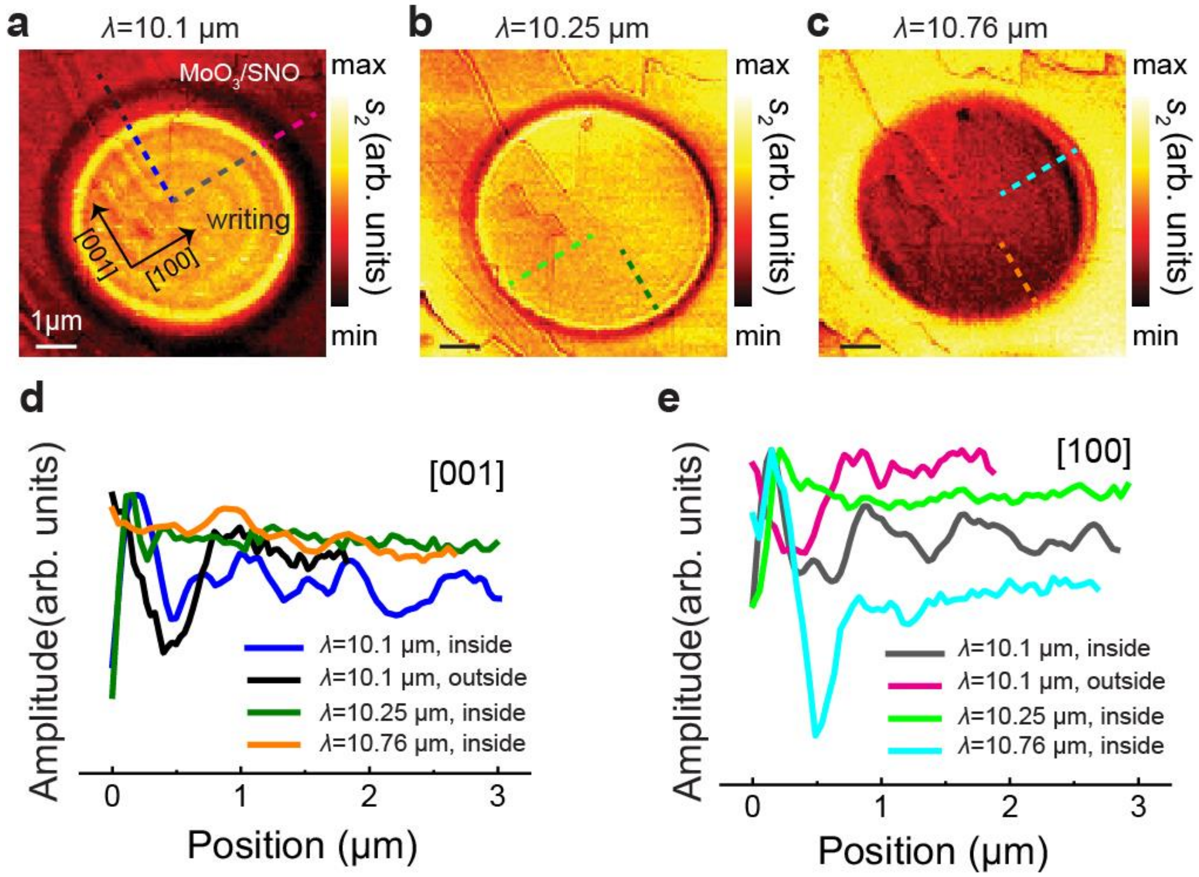

Supplementary Figure 12 Line-profiles on the near-field images in Fig. 5a-c of the manuscript. a-c The same near-field images as Fig. 5a-c but with broken lines added along the [100] and [001] axis. **d** Line profiles along the [001] and **e** along [100] axis in Fig. 5a-c.

Supplementary References

1 Ma, W. *et al.* In-plane anisotropic and ultra-low-loss polaritons in a natural van der Waals crystal. *Nature* **562**, 557-562 (2018). https://doi.org:10.1038/s41586-018-0618-9

2 Zheng, Z. *et al.* A mid-infrared biaxial hyperbolic van der Waals crystal. *Science Advances* **5**, eaav8690 (2019). https://doi.org:10.1126/sciadv.aav8690

3 Li, P. *et al.* Infrared hyperbolic metasurface based on nanostructured van der Waals materials. *Science* **359**, 892-896 (2018). https://doi.org:10.1126/science.aaq1704

4 Hu, G., Shen, J., Qiu, C.-W., Alù, A. & Dai, S. Phonon Polaritons and Hyperbolic Response in van der Waals Materials. *Advanced Optical Materials* **8**, 1901393 (2020). https://doi.org:https://doi.org/10.1002/adom.201901393

5 Li, P. *et al.* Collective near-field coupling and nonlocal phenomena in infrared-phononic metasurfaces for nano-light canalization. *Nature Communications* **11**, 3663 (2020). https://doi.org:10.1038/s41467-020-17425-9

6 Li, Z. Y. *et al.* Correlated Perovskites as a New Platform for Super-Broadband-Tunable Photonics. *Advanced Materials* **28**, 9117-+ (2016). https://doi.org:10.1002/adma.201601204

7 Dai, Z. *et al.* Artificial Metaphotonics Born Naturally in Two Dimensions. *Chemical Reviews* **120**, 6197-6246 (2020). https://doi.org:10.1021/acs.chemrev.9b00592
